# Emergency Medical Services resource capacity and competency amid COVID-19 in the United States: preliminary findings from a national survey

**DOI:** 10.1016/j.heliyon.2020.e03900

**Published:** 2020-05-03

**Authors:** Cody Gibson, Christian Ventura, George Donald Collier

**Affiliations:** a Council on Research and Continuing Education, Health Advocacy and Medical Exploration Society, Inc. Lawrenceville, NJ 08648 USA; b Department of Natural Sciences, Bard College at Simon's Rock, Great Barrington, MA 01230 USA; c Department of Natural Sciences, Calhoun Community College, Tanner, AL 35671 USA

**Keywords:** Emergency medicine, Health sciences, Infectious disease, Public health, EMS, Pre-hospital care, Novel coronavirus, COVID-19, SARS-CoV-2

## Abstract

**Objective:**

This study aimed to investigate available resources, Personal Protective Equipment (PPE) availability, sanitation practices, institutional policies, and opinions among EMS professionals in the United States amid the COVID-19 pandemic using a self-report survey questionnaire.

**Methods:**

An online 42-question multiple choice survey was randomly distributed between April 1, 2020, and April 16, 2020 to various active Emergency Medical Services (EMS) paid personnel in all 50 U.S. states including the District of Columbia (n = 192). We approximate a 95% confidence interval (±0.07).

**Results:**

An overwhelming number of EMS providers report having limited access to N95 respirators, receiving little or no benefits from COVID-19 related work, and report no institutional policy on social distancing practices despite CDC recommendations. For providers who do have access to N95 respirators, 31% report having to use the same mask for 1 week or longer. Approximately ⅓ of the surveyed participants were unsure of when a COVID-19 patient is infectious. The data suggests regular decontamination of EMS equipment after each patient contact is not a regular practice.

**Discussion:**

Current practices to educate EMS providers on appropriate response to the novel coronavirus may not be sufficient, and future patients may benefit from a nationally established COVID-19 EMS response protocol. Further investigation on whether current EMS practices are contributing to the spread of infection is warranted. The data reveals concerning deficits in COVID-19 related education and administrative protocols which pose as a serious public health concern that should be urgently addressed.

**Key Messages**

**What is already known on this subject**

• COVID-19 presents as a national emergency in the United States, and all efforts to mitigate the spread of disease should be considered
• Emergency Medical Services personnel play a pivotal role in patient outcomes and are often the first healthcare providers to make contact with COVID-19 patients
• The CDC has provided an Interim guidance for EMS professionals amid the COVID-19 pandemic

**What this study adds**

• Due to inconsistent decontamination practices and administrative protocols that are non-compliant with CDC recommendations, EMS providers may inadvertently contribute to the spread of infection
• Due to varied knowledge and opinions of EMS providers on COVID-19, current pandemic education approaches may need to be revisited

## 1 Introduction

As of April 16, 2020, SARS-CoV-2 has been responsible for approximately 632,548 infections and 31,071 deaths in the United States (CDC, 2020). Infection results in the development of coronavirus disease of 2019 (COVID-19) (WHO, 2020). EMS providers are potentially exposed to SARS-CoV-2 while providing patient care during the COVID-19 pandemic, yet data concerning Emergency Medical Services (EMS) providers and COVID19 is scarce. A search of NCBI using the terms “EMS AND COVID-19” returned no articles. Response to COVID-19 in the pre-hospital setting is currently guided by the Interim Guidance for Emergency Medical Services (EMS) Systems and 911 Public Safety Answering Points (PSAPs) for COVID-19 in the United States (CDC, 2020). This study aimed to investigate individual EMS provider competency and resource accessibility amid COVID-19 in the United States using a self-report survey questionnaire. Limitations for this study include a limited sample size and the consideration that no current model for this type of questioning exists.

## 2 Methods

An online 42-question multiple choice survey was created using a third party tool designed to collect data on individual provider demographics, institutional COVID-19 related practices, personal protective equipment (PPE) availability, and standard sanitation practices, along with a knowledge based section intended to assess COVID-19 related knowledge. The survey also featured a COVID-19 opinions section. Between April 1, 2020 and April 16, 2020 the survey was randomly distributed to various active Emergency Medical Services (EMS) personnel in all 50 U.S. states including the District of Columbia (n = 192) by way of multi-organizational distribution and promotion, press release, and social media distribution. An ethics approval was obtained and certified by the Institutional Review Board. The public or participants of this study were not involved in the design, conduct, reporting, or dissemination plans of research. Based on available U.S. Census data for EMS professionals, we approximate a 95% confidence interval with a marginal error of ±0.07 to reflect representation of practicing providers in the United States.

## 3 Results

**Figure 1:**
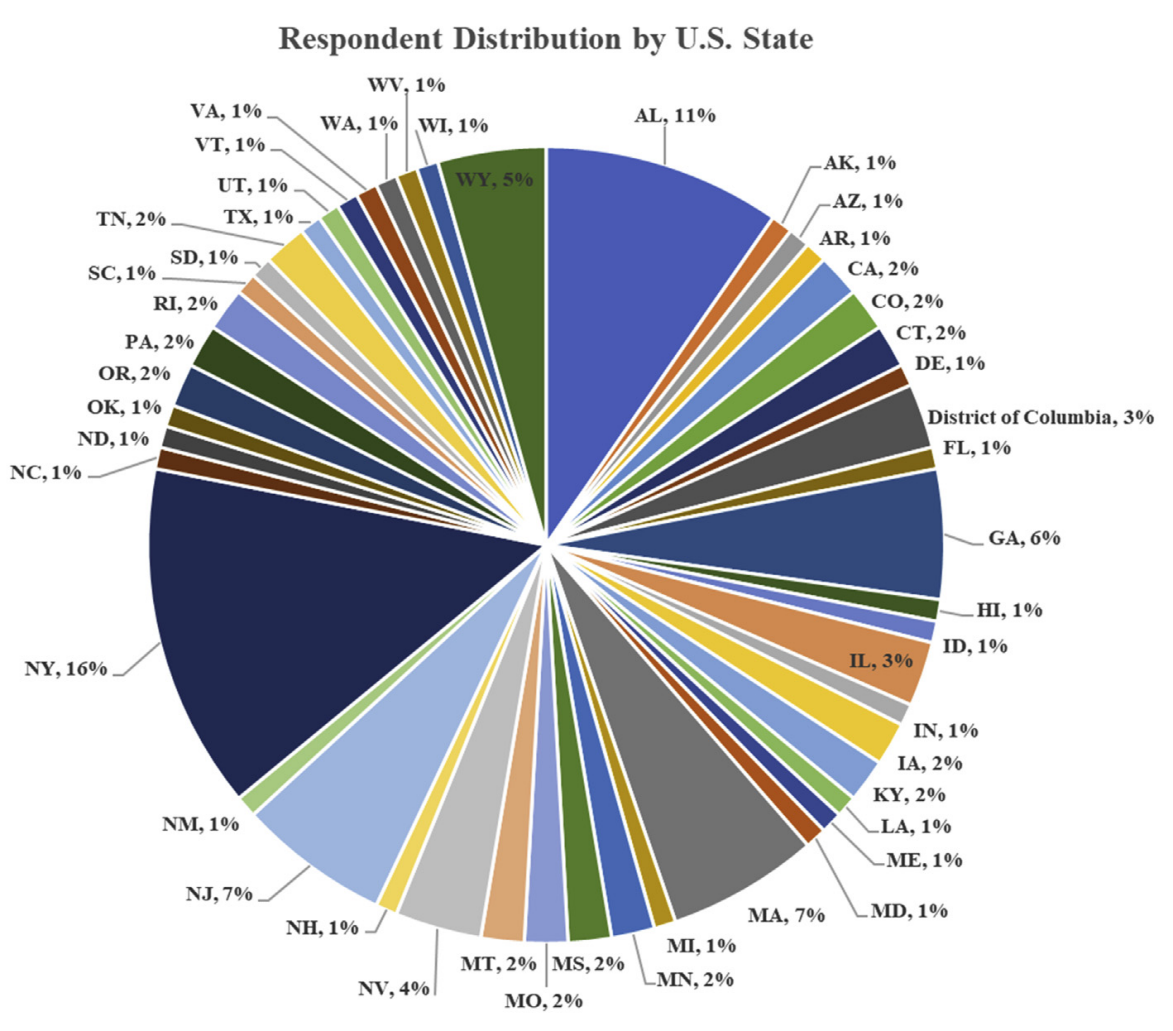
Respondent distribution by U.S. State.

As reflected in Table 1, the majority of survey respondents were EMT-Basics. However, the training level of respondents ranged from EMR to Paramedic. Most respondents reported that their education consisted of a high school diploma or GED. The minority of respondents reporting the earning of a graduate degree. The working environment of providers was reported to be mostly suburban. Figure 1 reflects respondent distribution by U.S. state.

**Table 1:** Demographics.

U.S. States represented: 47, including the District of Columbia Licensed provider level: - 10% Emergency Medical Responder (EMR) or equivalent - 61% Emergency Medical Technician Basic (EMT-B) or equivalent - 3% Emergency Medical Technician Intermediate (EMT-I) or equivalent - 7% Advanced Emergency Medical Technician (AEMT) or equivalent - 27% Paramedic (EMT-P) or equivalent - 2% EMS Registered Nurse - 1% Other Number of providers nationally registered: 63% Degree level held: - 5% Not applicable - 50% High school diploma, GED, or equivalent - 19% Associate's degree - 20% Bachelor's degree - 6% Masters degree Responding environment: - 47% Urban - 72% Suburban - 39% Rural - 21% Wilderness

As reflected in Table 2, early all EMS providers reported access to medical gloves when needed, with nitrile gloves being the most common gloves used in the pre-hospital setting. 48% of providers reported having access to N95 masks when needed. Of those who had access to N95 masks, 31% reported having to use the mask for 1 week or greater and 16% reported injury due to excessive PPE wear.

**Table 2:** PPE accessibility.

General PPE availability: - 94% of providers report access to medical gloves when needed - 48% of providers report access to N95 masks when needed If provided an N95 mask, providers report replacements are provided: - After each patient contact (15%) - After 3–5 patient contacts (5%) - After 1 day (7%) - After 3–5 days (11%) - After 1 week or greater (31%) 16% of providers report being injured due to excessive wear of PPE

As reflected in Table 3, 51% of EMS providers reported having limited training in COVID-19 response, with only 11% of EMS providers reporting extensive training, and EMS providers reported overall dissatisfaction with COVID-19 training with 18% reporting “Satisfied”, 34% “Neither satisfied nor dissatisfied”, 33% “Dissatisfied”, and 14% “Very dissatisfied”. With regard to benefits provided during response to COVID-19, 72% of EMS providers reported having no additional benefits. Time spent on COVID-19 related response was reported to be less than 5 hours per week for most EMS providers.

**Table 3:** COVID-19 related practices.

Reported type of COVID-19 related training: - Extensive training (11%) - Limited training (51%) - No training provided (36%) - Unsure (2%) Reported satisfaction with provided COVID-19 related training: - 18% Satisfied - 34% Neither satisfied nor dissatisfied - 33% Dissatisfied - 14% Very dissatisfied Reported types of benefits provided in response to COVID-19 related work: - None (70%) - Paid time off (4%) - Family and Medical Leave (9%) - Not applicable (23%) Average hours of the week dedicated to COVID-19: - Less than 5 hours (29%) - 5–7 hours (16%) - 7–10 hours (17%) - 10–15 hours (14%) - 15–20 hours (13%) - 20–40 hours (6%) - Greater than 40 hours (5%) Providers who report their employer would compensate them if quarantined: 30% Social distancing practices: - 33% report their facility recommends social distancing practices - 15% report their facility requires social distancing practices - 52% report their facility has no policy regarding social distancing practices Providers who would consider continuing to work and treat patients if tested positive for COVID-19 or experiencing COVID-19 like signs or symptoms: 36%

As reflected in Table 4, the majority of EMS providers report the use of nitrile gloves during regular EMS operations. 53% of providers reported decontaminating the patient compartment of their EMS unit after each patient contact, and 47% reported sanitizing their personal stethoscope after each patient contact. Disposable stethoscopes were reportedly widely unavailable to EMS facilities.

**Table 4:** Sanitation practices.

Types of gloves used: - Nitrile (96%) - Latex (4%) Types of disinfectants used for EMS unit: - Commercial industrial disinfectant (45%) - 1:10 Bleach solution (47%) - Other (8%) Frequency of EMS unit patient compartment decontamination: - 53% report after each patient contact - 35% report after multiple patient contacts - 12% report daily - 1% report infrequently Frequency of personal stethoscope decontamination: - 47% report after each patient contact - 1% report after multiple patient contacts - 9% report daily - 43% report infrequently Providers who report available access to disposable stethoscopes: 3%

As reflected in Table 5, EMS providers’ knowledge of COVID-19 PPE best practices and the presentation of signs and symptoms by patients following infection were assessed. The majority of EMS providers were able to differentiate between N95 masks and surgical masks. Most EMS providers reported that sneezing was not a sign of COVID-19 infection, COVID-19 is in the family of coronaviruses, the common cold is not an example of coronavirus, that one can contract COVID-19 inside of a 10 min timeframe of patient contact, and that patients can be asymptomatic and still infectious.

**Table 5:** COVID-19 knowledge.

The following statements were answered true or false by each provider: “N95 masks and surgical masks are the same.” - 2% report “True” - 83% report “False” - *Correct Response* - 15% report “Unsure” “A common symptom of COVID-19 is sneezing.” - 21% report “True” - 56% report “False” - *Correct Response* - 22% report “Unsure” “COVID-19 belongs to a family of coronaviruses.” - 73% report “True” - *Correct Response* - 6% report “False” - 21% report “Unsure” “The common cold is an example of a coronavirus.” - 28% report “True” - *Correct Response* - 52% report “False” - 20% report “Unsure” “Patient contact must be at least 10 minutes to catch COVID-19.” - 10% report “True” - 62% report “False” - *Correct Response* - 28% report “Unsure” “A patient is required to exhibit symptoms of COVID-19 to be infectious.” - 4% report “True” - 77% report “False” - *Correct Response* - 20% report “Unsure”

As reflected in Table 6, the majority of providers are in agreement that the novel coronavirus is worse than the flu, however there are highly varied opinions on media portrayal of COVID-19. The majority of providers neither agree or disagree that they are at increased risk for severe illness due to COVID-19 exposure.

**Table 6:** COVID-19 OPINIONS.

The following statements were ranked in agreement by each provider: “The novel coronavirus is worse than the flu” - 36% report “Strong agree” - 41% report “Agree” - 19% report “Neither agree nor disagree” - 4% report “Disagree” - 1% report “Strongly disagree” “COVID-19 is not as bad as the media portrays” - 2% report “Strong agree” - 22% report “Agree” - 27% report “Neither agree nor disagree” - 32% report “Disagree” - 18% report “Strongly disagree” “I am at increased risk for severe illness due to exposure to COVID-19” - 19% report “Strong agree” - 23% report “Agree” - 37% report “Neither agree nor disagree” - 19% report “Disagree” - 2% report “Strongly disagree” “My facility is or was prepared to respond effectively to the COVID-19 pandemic” - 1% report “Strong agree” - 29% report “Agree” - 20% report “Neither agree nor disagree” - 34% report “Disagree” - 16% report “Strongly disagree”

## 4 Discussion

Evidently, further investigation is warranted, most notably an increase in sample size. The limitations of this study predominantly revolve around marginal error. The preliminary data suggests, however, that providers may benefit from improved standardized training in pandemic response, specifically with regard to clinical symptomatology recognition, origins of the disease, a uniformed decontamination protocol, pandemic-specific inventory inservice, and stricter regulations and enforcement on decontamination of personal items, such as stethoscopes. The employment of disposable stethoscopes for EMS providers may also prove beneficial in reducing spread of infection. The data also warrants investigation on the efficacy of currently practiced decontamination procedures, as well as the presence of pathogenic novel coronavirus on the surface area of EMS equipment.

EMS providers appear to have differing views on whether they are at an increased risk for severe illness, despite current research that suggests healthcare providers are at a significant risk (Ng K et al., 2020; Zou L et al., 2020). Additionally, almost one-third of surveyed providers report being unsure whether a COVID-19 patient is infectious, and more than one-half of participants inaccurately identified a common symptom of COVID-19. Current practices to appropriately educate EMS providers on the novel coronavirus may not be sufficient, and families of providers and future patients may benefit from a nationally established COVID-19 EMS response protocol that complements or supersedes the recommendation of the current Interim Guidance by the CDC. The data reveals concerning deficits in COVID-19 related education and administrative protocols which potentially poses a serious public health concern that should be urgently addressed to reduce the spread of infection.

## Declarations

### Author contribution statement

C. Gibson and C. Ventura: Conceived and designed the experiments; Performed the experiments; Analyzed and interpreted the data; Wrote the paper.

G. Collier: Performed the experiments; Analyzed and interpreted the data.

**Research Assistants:** Hyewon Sabrina Baang^2^, Edward Denton^1,2^, Christopher Knauth^2^, Emily Van Court^1,2^, Maya Saraya^2^, Paul Kameen^2^

### Funding statement

This research did not receive any specific grant from funding agencies in the public, commercial, or not-for-profit sectors.

### Competing interest statement

The authors declare no conflict of interest.

### Additional information

No additional information is available for this paper.
