# Corrigendum to “Emergency Medical Services resource capacity and competency amid COVID-19 in the United States: preliminary findings from a national survey” [Heliyon 6 (5) (May 2020) e03900]

**DOI:** 10.1016/j.heliyon.2020.e05409

**Published:** 2020-11-02

**Authors:** Cody Gibson, Christian Ventura, George Donald Collier

**Affiliations:** a Council on Research and Continuing Education, Health Advocacy and Medical Exploration Society, Inc. Lawrenceville, NJ 08648, USA; b Department of Natural Sciences, Bard College at Simon's Rock, Great Barrington, MA 01230, USA; c Department of Natural Sciences, Calhoun Community College, Tanner, AL 35671, USA

In the original published version of this article, one affiliation (Council on Research and Continuing Education, Health Advocacy and Medical Exploration Society, Inc) was incorrectly attributed to author Cody Gibson. This has now been corrected. Additionally, the corresponding authorship has now been extended to all three authors, Cody Gibson, Christian Ventura and George Donald Collier to allow for representation and correspondence from each authors' institutions. The authors apologize for these mistakes. Both the HTML and PDF versions of the article have been updated to correct the errors.

